# Sorbitol promotes the graft healing process in pears

**DOI:** 10.1093/hr/uhaf168

**Published:** 2025-06-25

**Authors:** Jianlong Liu, Baoyi Wang, Siying Zhang, Min Liu, Wankun Liu, Ping Yang, Chenglin Liang, Jiankun Song, Yingjie Yang, Ran Wang, Dingli Li

**Affiliations:** Qingdao Key Lab of Genetic Improvement and Breeding of Horticultural Plants, College of Horticulture, Qingdao Agricultural University, No. 700, Changcheng Road, Chengyang District, Qingdao City, Shandong Province 266109, China; Qingdao Key Lab of Genetic Improvement and Breeding of Horticultural Plants, College of Horticulture, Qingdao Agricultural University, No. 700, Changcheng Road, Chengyang District, Qingdao City, Shandong Province 266109, China; Qingdao Key Lab of Genetic Improvement and Breeding of Horticultural Plants, College of Horticulture, Qingdao Agricultural University, No. 700, Changcheng Road, Chengyang District, Qingdao City, Shandong Province 266109, China; Qingdao Key Lab of Genetic Improvement and Breeding of Horticultural Plants, College of Horticulture, Qingdao Agricultural University, No. 700, Changcheng Road, Chengyang District, Qingdao City, Shandong Province 266109, China; Qingdao Key Lab of Genetic Improvement and Breeding of Horticultural Plants, College of Horticulture, Qingdao Agricultural University, No. 700, Changcheng Road, Chengyang District, Qingdao City, Shandong Province 266109, China; Qingdao Key Lab of Genetic Improvement and Breeding of Horticultural Plants, College of Horticulture, Qingdao Agricultural University, No. 700, Changcheng Road, Chengyang District, Qingdao City, Shandong Province 266109, China; Haidu College, Qingdao Agricultural University, No. 11, Wenhua Road, Laiyang City, Shandong Province, 265200, China; Qingdao Key Lab of Genetic Improvement and Breeding of Horticultural Plants, College of Horticulture, Qingdao Agricultural University, No. 700, Changcheng Road, Chengyang District, Qingdao City, Shandong Province 266109, China; Qingdao Key Lab of Genetic Improvement and Breeding of Horticultural Plants, College of Horticulture, Qingdao Agricultural University, No. 700, Changcheng Road, Chengyang District, Qingdao City, Shandong Province 266109, China; Academy of Dongying Efficient Agricultural Technology and Industry on Saline and Alkaline Land in Collaboration with Qingdao Agricultural University, No. 01, Gaoxin Road, Dongying City, Shandong Province, 257091, China; Qingdao Key Lab of Genetic Improvement and Breeding of Horticultural Plants, College of Horticulture, Qingdao Agricultural University, No. 700, Changcheng Road, Chengyang District, Qingdao City, Shandong Province 266109, China; Qingdao Key Lab of Genetic Improvement and Breeding of Horticultural Plants, College of Horticulture, Qingdao Agricultural University, No. 700, Changcheng Road, Chengyang District, Qingdao City, Shandong Province 266109, China; Academy of Dongying Efficient Agricultural Technology and Industry on Saline and Alkaline Land in Collaboration with Qingdao Agricultural University, No. 01, Gaoxin Road, Dongying City, Shandong Province, 257091, China

## Abstract

Pear propagation is primarily achieved through asexual reproduction via grafting. During the graft union healing process, there is metabolic exchange between the rootstock and the scion. However, a multi-omics systematic study on the role of sugar in the graft union healing process has not been reported. In this study, using micrografting techniques, we comparatively analyzed the metabolic changes during the healing process in homograft and heterograft of pear through metabolomics and transcriptomics. We found significant differences in sugar metabolism pathways after grafting. In the fructose and mannose metabolic pathways, sorbitol exhibited opposite trends in homograft and heterograft. Subsequent transcriptomics analysis confirmed that these metabolite changes were caused by differential expression of related synthetic and converting enzyme genes. Furthermore, spatial metabolomics identified sorbitol accumulation in the scion after homologous grafting. To further verify the role of sorbitol, exogenous sorbitol treatment was applied, revealing that it enhanced tissue adhesion, shortened the time required for callus growth, promoted high expression of xylem formation genes and cambium differentiation genes, and facilitated the reconnection of xylem and phloem, thereby playing a positive role in graft union healing. This study systematically analyzed changes in sugar metabolism during the grafting process and confirmed that sorbitol can promote graft union healing.

## Introduction

Pears have a long cultivation history and are widely planted worldwide with numerous varieties. Grafting, as an efficient horticultural technique that can shorten the juvenile phase, enhance resistance to biotic or abiotic stresses, optimize flowering habits, and improve plant architecture, is widely used in pear industry production and seedling propagation [1–4]. Graft union healing and grafting compatibility have always been research hotspots in botany and life sciences.

Generally, there are large gaps and apoptotic cells between the rootstock and scion at the initial stage of grafting. Subsequently, the wounded cells are triggered to secrete pectin, which adheres the rootstock and scion together. The dedifferentiated stem cell tissue, known as callus, begins to fill the space between the rootstock and scion and connects them. The cambium, cortex, and pith cells around the phloem, along with the callus, differentiate to form a complete vascular tissue, with the phloem connection preceding the xylem connection [5–11]. The key step for successful grafting is the reconnection of functional vascular bundle tissues. Graft incompatibility is caused by fewer vascular bundle tissue connections and tissue asymmetry resulting from phloem degeneration [12, 13]. What affects the later differentiation and connection is the arrangement of callus cells and differences in the content of metabolic substances such as cellulose, lipids, and phenolics [14, 15]. It can thus be seen that the accumulation of different metabolites during the grafting process plays a crucial role in graft healing. Currently, researchers are analyzing changes in various metabolites to screen and identify their impacts on graft healing, with a particular focus on sugar metabolism. Present research has revealed that sugars can promote cell division and cell expansion, and sugar metabolism is also implicated in the graft healing process [6, 16]. Soluble sugars and various amino acids accumulated at the graft union 33 days after grape grafting [17]. Starch accumulated above the cut surface in grafted Arabidopsis, and this accumulation decreased as asymmetry disappeared once the vascular tissues reconnected [18]. Micrografting with low concentrations of sucrose added to the medium results in higher grafting success rates [19]. Similar results confirmed that sucrose can promote the survival rate of grafted plants [20, 21]. Meanwhile, the central process of sugar metabolism in all cells is the sucrose cycle, and in Rosaceae fruit tree species such as apple and pear, sorbitol is the main end product of photosynthesis [22]. The role of sorbitol in the grafting process of pear is unknown. Compared to sugars, sugar alcohols exhibit more favorable characteristics when plants are coping with unfavorable environmental conditions. Sorbitol can improve plant salt tolerance, drought resistance, and enhance fruit cold tolerance [23–25]. The addition of sorbitol in plant tissue culture can significantly enhance the differentiation and regeneration capacity of callus tissue [26]. In higher plants, sorbitol can be metabolized into fructose or glucose. Furthermore, changes in sorbitol content in specific tissues are closely related to the long-distance transport of sorbitol, and stress-induced changes in sorbitol metabolism (synthesis and degradation rates) are the main reasons for regulating sorbitol levels in plant tissues [27]. Therefore, it is urgent to elucidate the changes in sorbitol metabolism during pear grafting and its role in grafting.

**Figure 1:**
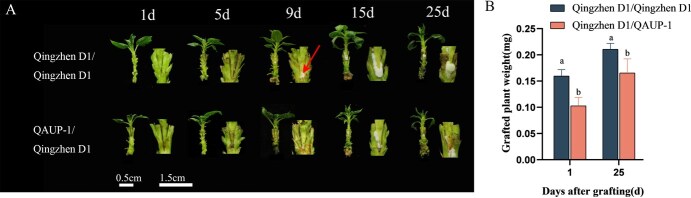
Growth performance analysis of different graft combinations. (A) Phenotypic diagram of tissue culture grafted seedlings of ‘Qingzhen D1/Qingzhen D1’ and ‘QAUP-1/Qingzhen D1’. Bar = 0.5 cm for plant, bar = 1.5 cm for grafting site. (B) Grafted plant weigh of ‘Qingzhen D1/Qingzhen D1’ and ‘QAUP-1/Qingzhen D1’. Different letters above the bars indicate significant differences (Duncan’s multiple range test *P* < 0.05).

With the advancement of bioinformatics, many studies are utilizing metabolomics, transcriptomics, and other approaches to investigate substance changes during the grafting process, aiming to identify key metabolites that promote healing. For instance, metabolomics analysis revealed that light could regulate phenylpropanoid biosynthesis, cysteine and methionine metabolism, and amino sugar and nucleotide sugar metabolism, thereby facilitating graft healing through cell wall synthesis [28]. Transcriptome and metabolome analyses unveiled the impact of hormones on the growth of scions grafted onto different rootstocks within the same year [29]. However, these studies have been unable to accurately identify the spatial distribution of key metabolites. Spatial metabolomics, which combines traditional metabolomics techniques with mass spectrometry (MS) imaging technology, can address this issue. It not only identifies which substances are present in the sample and their quantities but also detects the spatial distribution information of compounds in a single experiment, achieving both qualitative and quantitative localization. This approach has already been applied to investigate metabolic changes during the fruit development of horticultural crops such as strawberries and tomatoes, identifying the spatial distribution of metabolites like citric acid and anthocyanins during development and confirming their correlations with fruit traits such as color [30–32]. However, no relevant reports have been found in studies investigating the healing process of fruit tree grafting that utilize spatial metabolomics approaches.

Therefore, in this study, different grafting combinations were used to comparatively analyze the material changes during the healing process. Metabolomics and transcriptome analysis were employed to identify the conversion of sugar metabolism at the graft union. Spatial metabolome analysis was used to determine the spatial distribution of sorbitol in the rootstock and scion. Additionally, histological observations, cytological observations, and gene expression analysis under sorbitol induction were conducted to determine the role of sorbitol in the healing process of pear grafts. The aim is to provide a theoretical basis and technical guidance for the rapid healing of pear grafts.

## Results

### Effects of self-grafting and heterografting on graft union healing

Both self-grafting and heterografting demonstrated successful healing, with a 100% graft survival rate. However, the scions of the ‘Qingzhen D1/Qingzhen D1’ grafting combination exhibited broad and stretched-out leaves, and the plant growth vigor was significantly superior to that of the ‘QAUP-1/Qingzhen D1’ combination. The formation of callus in the ‘Qingzhen D1/Qingzhen D1’ grafting combination occurred earlier than in the ‘QAUP-1/Qingzhen D1’ combination, with noticeable callus appearing on the ninth day. In contrast, the heterologous grafted combination (QAUP-1/Qingzhen D1) only showed distinct callus formation until the 15th day after grafting. This indicates that the self-grafted combination (Qingzhen D1/Qingzhen D1) exhibited better growth after grafting compared to the heterologous grafted combination (Fig. 1A). But after 25 days of grafting, there was no significant difference in biomass between the ‘QAUP-1/Qingzhen D1’ grafting combination and the ‘Qingzhen D1/Qingzhen D1’ combination (Fig. 1B).

### Transcriptomic and metabolomic profiling of carbohydrate metabolism dynamics during graft union healing

**Figure 2:**
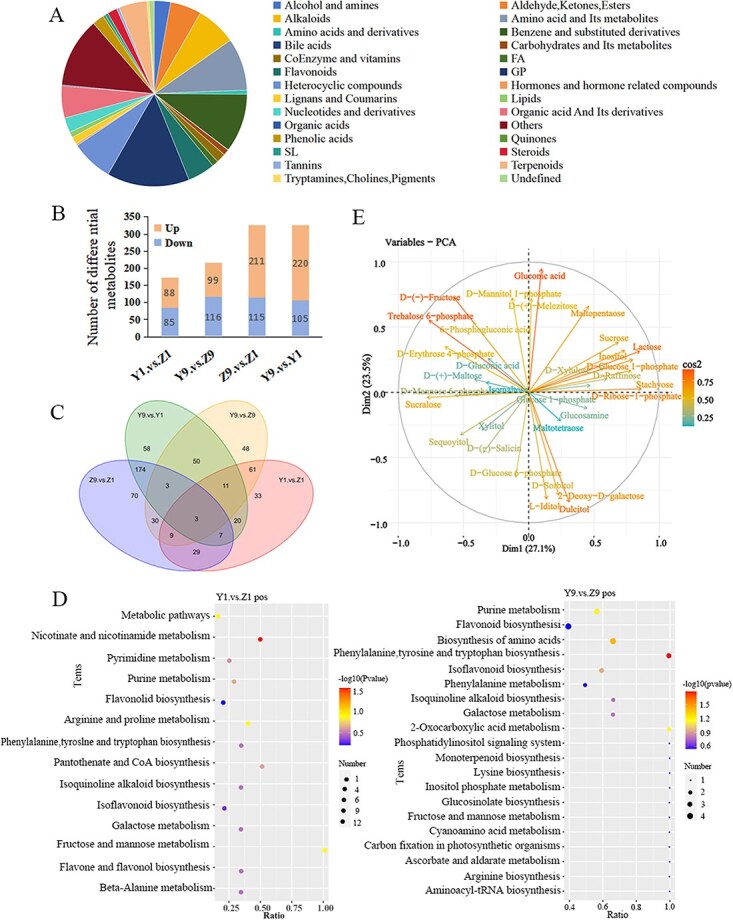
Analysis of metabolomics data in heterografts and homografts combination. (A) Categorical analysis of total metabolites. (B) Bar chart of the percentage of period-specific differential metabolites (DEMs) in heterografts and homografts. (C) Venn diagram showing the number of DAMs identified in the comparison of Z9 vs Z1, Y9 vs Y1, Y9 vs Z9, and Y1 vs Z1, (D) KEGG enrichment analysis of the differences in the Y1 vs Z1 and Y9 vs Z9 metabolic pathways, (E) Glycan metabolite PCA loading diagram. ‘Count’ refers to the number of DEMs annotated to the KEGG pathway, and ‘Gene Ratio’ refers to the ratio of the number of DEGs annotated to a KEGG pathway to the total number of DEGs annotated to all KEGG pathways. Z1: Day 1 after homografting, Z9: Day 9 after homografting, Y1: Day 1 after heterografting, and Y9: Day 9 after heterografting.

To better understand the dynamic changes in related metabolites during the grafting healing process, metabolite identification was conducted through ultra-performance liquid chromatography–mass spectrometry (UPLC–MS) analysis of two grafting combinations at 1 and 9 days postgrafting. A total of 1594 metabolites were identified and annotated (Fig. 2A), among which 173 differential metabolites were detected between Y1 and Z1, including 88 upregulated and 85 downregulated metabolites, with 33 specific differential metabolites. Between Y9 and Z9, a total of 215 differential metabolites were detected, with 99 upregulated and 116 downregulated metabolites, and 48 specific differential metabolites (Fig. 2B and C).

Kyoto Encyclopedia of Genes and Genomes (KEGG) annotation analysis was performed on the identified differential metabolites. The main enriched pathways for the differential metabolites in Y1 vs Z1 were ‘Biosynthesis Pathways’, ‘Lysine Biosynthesis’, ‘Glycine, Serine, and Threonine Metabolism’, ‘Flavonoid Biosynthesis’, and ‘Fructose and Mannose Metabolism’. For Y9 vs Z9, the primary enriched pathways were ‘Fructose and Mannose Metabolism’, ‘Galactose Metabolism’, ‘Flavonoid Biosynthesis’, as well as several ‘Secondary Metabolism’, ‘Terpenoids’, and ‘Phenolic Compounds’ pathways (Fig. 2D). Principal component analysis (PCA) of carbohydrate metabolism revealed that the main carbohydrate metabolites at the grafting interface of the self-grafted and heterologous grafted combinations were dispersed and enriched at the positive and negative poles of Dim1 and Dim2. Generally, fructose (D-Fructose), trehalose 6-phosphate (Trehalose 6-phosphate), sucrose (Sucrose), sorbitol (D-Sorbitol), dulcitol (Dulcitol), and galactitol were abundant at the grafting site, while D-mannose 6-phosphate, isomaltose, and xylitol were less abundant (Fig. 2E).

### Integrated transcriptomic and metabolomic analysis of sucrose and sorbitol metabolic pathways

**Figure 3:**
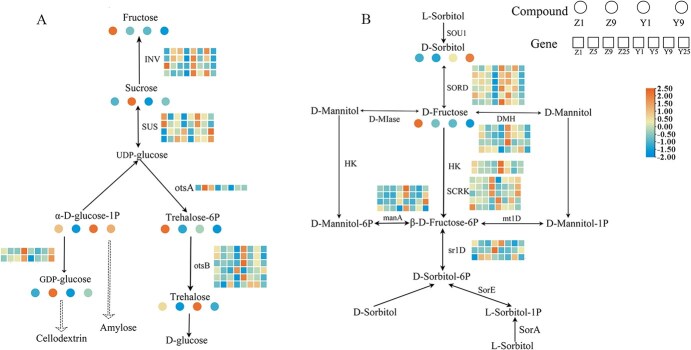
Analysis of carbohydrate changes during the healing process of heterograft and homograft combinations. (A) Starch and sucrose pathways. (B) Mannose and fructose pathways. Z1: Day 1 after homografting, Z5: Day 5 after homografting, Z9: Day 9 after homografting, Z25: Day 25 after homografting, Y1: Day 1 after heterografting, Y5: Day 5 after heterografting, Y9: Day 9 after heterografting, Y25: Day 25 after heterografting.

To further elucidate the changes in carbohydrate metabolism during the grafting process, key metabolites and related enzyme genes in the starch and sucrose pathway, as well as the mannose and fructose metabolism pathway, were screened through metabolome and transcriptome analysis. The results showed that regardless of the grafting combination, the relative contents of fructose, trehalose, trehalose 6-phosphate, and GDP-glucose generally decreased as the grafting healing progressed. In contrast, the relative contents of sucrose and glucose phosphate generally increased, but both were higher in the self-grafted combination than in the heterologous grafted combination. Neutral invertase, which catalyzes the conversion of fructose to sucrose, was highly expressed in the self-grafted combination on the fifth and ninth days postgrafting, compared to the heterologous grafted combination (Fig. 3A, Supplementary Table S1). In the ‘mannose and fructose metabolism pathway’, it is noteworthy that sorbitol exhibited differential expression in the two grafting combinations, decreasing in the self-grafted combination and increasing in the heterologous grafted combination. The sorbitol dehydrogenase gene, which catalyzes the decomposition of sorbitol to fructose, was highly expressed in the heterologous grafted combination. The 6-phosphosorbitol dehydrogenase gene was initially downregulated on the fifth and ninth days postgrafting but then upregulated on the 25th day, regulating sorbitol synthesis. The aldose reductase gene, which regulates sorbitol synthesis, was downregulated throughout the entire grafting and growth period (Fig. 3B).

### Spatial metabolomic profiling of sorbitol distribution during graft union healing

**Figure 4:**
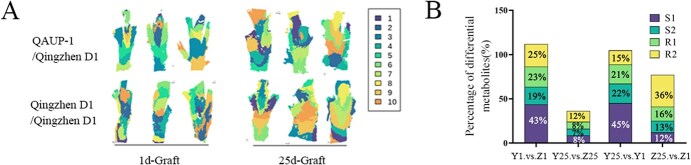
Analysis of metabolites in spatial locations in heterografts and homografts. (A) Spatially contracted center-of-mass clustering plots of heterografts and homografts combinations. (B) Bar charts of the percentage of different regional DEMs. S1: The upper part of the scion, S2: The lower part of the scion, R1: The upper part of the rootstock, R2: The lower part of the rootstock

To further investigate the spatial distribution of sugars and their metabolites, we conducted spatial metabolome sequencing and constructed a data-driven tissue section segmentation map based on regional-specific metabolite profiles. By grouping similar mass spectra into the same category and marking them with the same color, we identified a total of 10 distinct clusters. The different colors in the figure represent different regions of spatial segmentation (Fig. 4A, Supplementary Fig. S1). One day after grafting, the scion and rootstock in heterologous grafts exhibited clear layering and significant color contrasts, indicating large differences in internal metabolites. In contrast, in self-grafts, there was overlap in the color coverage areas of the scion and rootstock, suggesting a degree of similarity. Twenty-five days after grafting, self-grafts displayed minimal color contrast among adjacent gradients, with a fusiform progression starting from the scion and extending toward the rootstock. This morphology was fully consistent with the grafting method employed. Mass spectrum classification through spatial contraction centroid clustering was performed on individual samples. To better screen for regional characteristic metabolites that affect grafting healing, we conducted a more detailed regional division of the overall grafted tissue. Region S1 represented the upper part of the scion, S2 the lower part of the scion, R1 the upper part of the rootstock, and R2 the lower part of the rootstock (Supplementary Fig. S2). When comparing self-grafts and heterologous grafts on the first day, we found that 43% of differential metabolites were in region S1, 19% in S2, 23% in R1, and 25% in R2. On the 25th day, there was no significant difference in the number of differential metabolites among the regions, indicating that as grafting healing progressed, differences within the tissue gradually decreased (Fig. 4B).

KEGG enrichment analysis of the differential metabolites revealed that, on the first day after grafting, in the comparison of the upper scion between self-grafts and heterologous grafts (Y1-S1 vs Z1-S1), most metabolites in amino acid pathways such as ‘Secondary metabolite biosynthesis’, ‘Cysteine and methionine metabolism’, and ‘Valine, leucine, and isoleucine degradation’ showed a decreasing trend, while metabolites in pathways such as ‘Carbon fixation in photosynthetic organisms’ and ‘alpha-Linolenic acid metabolism’ increased. At the grafting interface, differential metabolites in regions S2 and R1 were enriched in carbon cycle metabolic pathways such as ‘Fructose and mannose metabolism’, ‘Starch and sucrose metabolism’, and ‘Galactose metabolism’ (Supplementary Figs S3 and S4).

**Figure 5:**
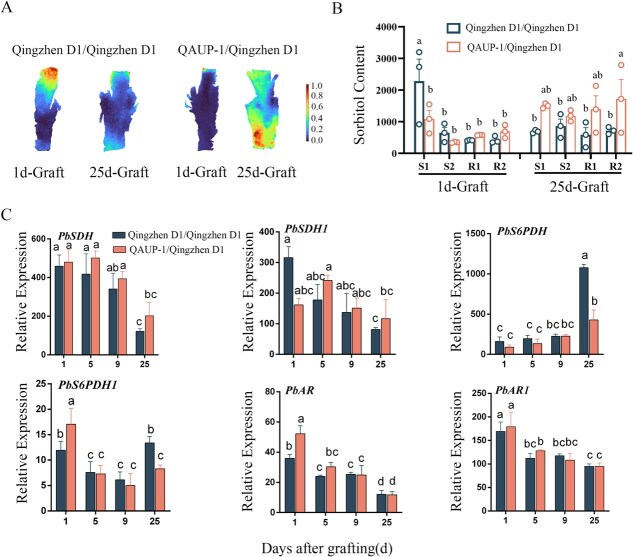
Analysis of spatial distribution, content dynamics, and related gene expression of sorbitol during the graft healing process. (A) Distribution of sorbitol content in spatial regions. (B) Effect of nongrafting combinations on sorbitol content, S1: The upper part of the scion; S2: The lower part of the scion; R1: The upper part of the rootstock; R2: The lower part of the rootstock. (C) Expression of genes related to sorbitol synthesis metabolism. Different letters above the bars indicate significant differences (Duncan’s multiple range test *P* < 0.05).

The spatial distribution map shows that after 1 day of grafting, compared with QAUP-1/Qingzhen D1, the expression of sorbitol in the upper part of the scion is significantly upregulated in the Qingzhen D1/Qingzhen D1 combination. And after 25 days of grafting, the expression of sorbitol in both the scion and rootstock of QAUP-1/Qingzhen D1 combination showed significant upregulation (Fig. 5A). In heterologous grafts, the sorbitol content exhibited an increasing trend, with significant increases observed particularly in regions R1 and R2. In self-grafts, sorbitol levels were higher in regions S1 and S2 1 day after grafting, but decreased in all regions 25 days after grafting (Fig. 5B). In order to further investigate the metabolic changes of sorbitol, the expression levels of genes involved in sorbitol synthesis and degradation were analyzed. The results indicate that compared to self-grafting, the expression of *PbSDH* and *PbSDH1* was increased after heterologous grafts, with *PbS6PDH1* and *PbAR* being significantly upregulated on the first day after grafting (Fig. 5C).

### Sorbitol promotes graft union healing

**Figure 6:**
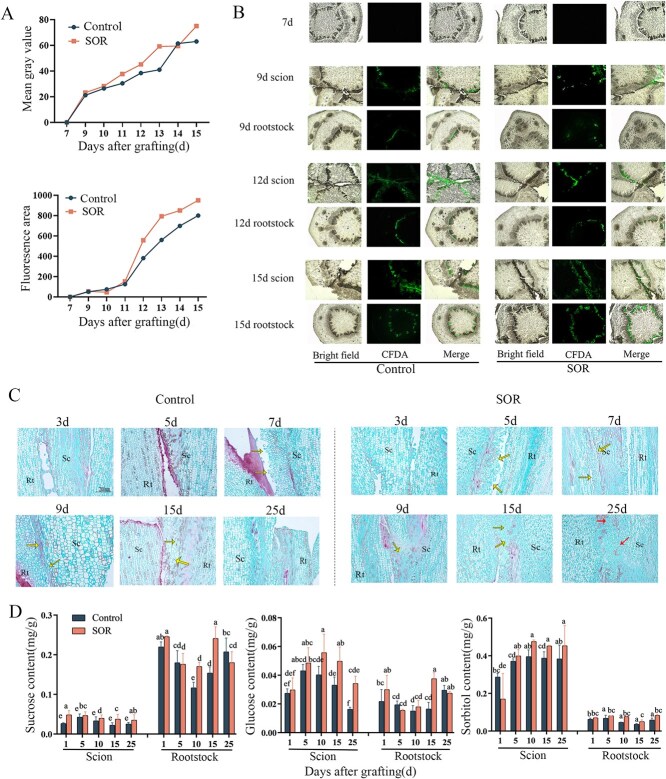
Morphological observation of graft union tissue promoted by sorbitol. (A) Average fluorescence intensity and fluorescence region. (B) CFDA fluorescence tracer plot. (C) Microscopic observation of grafting tissue treated with sorbitol. In the graft junction of the sorbitol-treated group at 25 days, the arrow marks the vascular bundle. For the remaining parts of the graft junction, the arrow marks the callus tissue. Sc: scion; Rt: rootstock. (D) Sugar content chart. Different letters above the bars indicate significant differences (Duncan’s multiple range test *P* < 0.05). Control: control group; SOR: Sorbitol treatment group.

To investigate whether sorbitol plays a role in the process of vascular reconnection, we employed carboxyfluorescein diacetate (CFDA) labeling to detect fluorescent signals from viable cells in cross-sections at the graft interface and the stock (5 cm below the graft interface). By comparing the average fluorescence intensity and fluorescence area data, it was found that compared with the control group, the fluorescence intensity of the group receiving exogenous sorbitol application was significantly enhanced 9 days after treatment; after 11 days of treatment, the fluorescence area significantly increased (Fig. 6A). Notably, 7 days after grafting, no fluorescent signals were observed in any vascular tissues. Nine days postgrafting, weak fluorescence emerged in both the control and treated groups. By 12 days postgrafting, the fluorescence at the graft interface cross-section had spread to the surrounding parenchyma cells. Fifteen days after grafting, a strong fluorescent signal was detectable in the stock (Fig. 6B).

To ascertain the role of exogenous sorbitol during various critical stages of the grafting healing process, anatomical observations were conducted on the samples. On the 5th day postgrafting, due to damage to parenchyma cells at the junction between the stock and scion, a visible condensation of protoplasm occurred, forming an isolating layer on the wound surface. In the xylem parenchyma cells and rays of the scion that were not treated with sorbitol, dedifferentiated cells began to radially thicken and protrude outward, with a significant increase in parenchyma cells that were irregularly piled up. Parenchyma cells were larger and clearly contained larger nuclei, with the isolating layer being distinctly visible. In contrast, after sorbitol treatment, obvious callus had already formed and was proliferating extensively around, forming clusters of callus cells in a disordered arrangement. On the seventh day postgrafting, the control scion also differentiated to form callus, albeit later than in the sorbitol-treated scion and stock. Nine days postgrafting, the proliferation and connection of callus at the graft union filled the gap between the stock and scion due to the encapsulation and connection of the callus. By the 15th day, the two were tightly bound, forming a callus bridge, while the isolating layer had disappeared. At this point, the callus bridge became the main pathway for cellular communication between the stock and scion. On the 25th day postgrafting, newly differentiated vascular bundles in the sorbitol-treated group expanded and differentiated, presenting an irregular distribution. Vessels within the mixed vascular bundle population were clearly visible, indicating that the grafted plant had formed a new individual (Fig. 6C).

Afterwards, in order to determine whether sorbitol treatment had an effect on the sugar content of pear-grafted seedlings, the sugar content of grafted seedlings was measured. The results showed that after 10–25 days of treatment with sorbitol, the sorbitol content in the scions was significantly higher than that in the control group; the glucose content in the scions treated with sorbitol was significantly higher than that in the control group. After 15 days of grafting, the glucose content in the rootstock treated with sorbitol increased; after treatment with sorbitol, the sucrose content in the rootstock is affected. On the 10th day after grafting, the sucrose content in the rootstock was higher than that in the control group (Fig. 6D).

**Figure 7:**
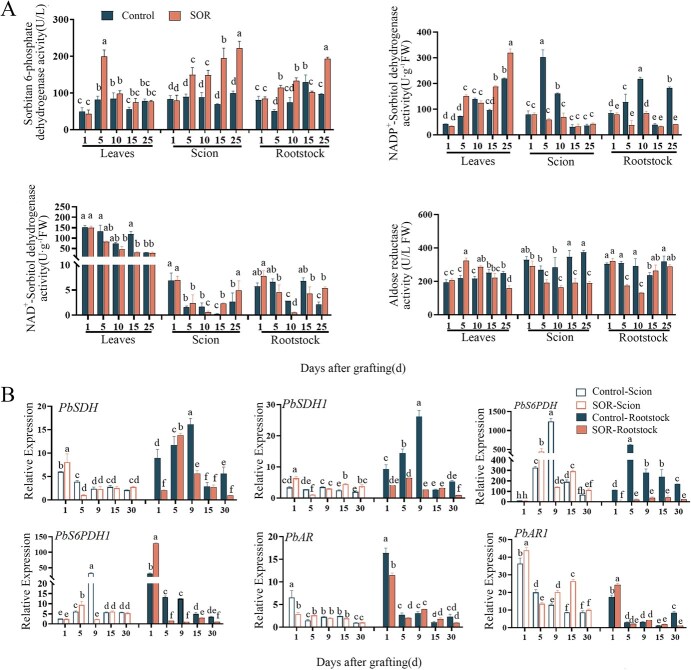
Analysis of sorbitol-treated anabolic enzyme activity and gene expression. (A) Enzyme activity of sorbitol anabolic pathway, Control: control group; SOR: Sorbitol treatment group. (B) Gene expression of enzymes related to sorbitol anabolic pathway. Different letters above the bars indicate significant differences (Duncan’s multiple range test *P* < 0.05)

The activities of sorbitol-related metabolic enzymes during graft union healing were examined. The results showed that the activities of sorbitol-6-phosphate dehydrogenase (S6PDH) and aldose reductase (AR) significantly increased 1 day after grafting. In contrast, the activities of NADP^+^-dependent sorbitol dehydrogenase (NADP^+^-SDH) and NAD^+^-dependent sorbitol dehydrogenase (NAD^+^-SDH) significantly decreased 1 day after grafting but showed an upward trend 10 days after grafting (Fig. 7A).

To further investigate the metabolic changes of sorbitol, the expression levels of sorbitol synthesis genes (S6PDH, AR) and degradation genes (SDH) were analyzed. The results indicated that SDH was significantly downregulated 5 days after grafting in both heterologous and self-grafts. S6PDH showed a significant upregulation trend 9 days after grafting and its expression was higher in self-grafts than in heterologous grafts. AR exhibited a significant downregulation trend 1 day after grafting. When exogenous sorbitol was applied, SDH was significantly expressed in the rootstock, while S6PDH was significantly expressed in the scion (Fig. 7B).

**Figure 8:**
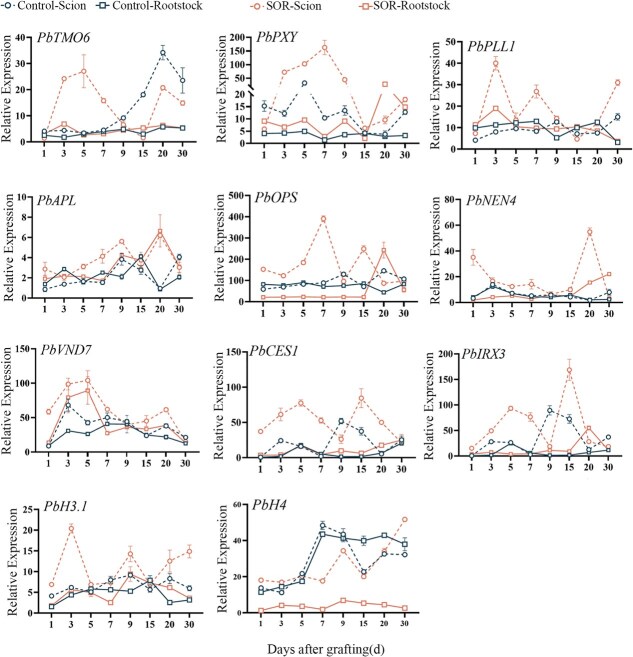
Transcription dynamics of genes related to vascular reconnection. Control: control group; SOR: sorbitol treatment group.

By analyzing the gene expression profiles related to vascular bundle formation and cell division, it was found that the positive regulatory factors of the phloem were activated 1 day after grafting and reached their peak expression levels at 7 days. Exogenous sorbitol treatment was able to enhance the midphase expression of *PbOPS* and promote high expression of *PbNEN4* in the later stages. Sorbitol also increased the relative expression levels of the xylem regulatory factors *PbVND7*, *PbIRX3*, and *PbCESA4* in grafted seedlings, showing a significant enhancement trend starting from 1 day after grafting. However, the expression levels significantly decreased at 7 and 9 days after grafting, and two expression peaks appeared throughout the healing period. Additionally, some genes related to the vascular bundle and cambium, such as *PbTMO6*, *PbPXY*, and *PbPLL1*, were activated and expressed in the early stages. The application of sorbitol significantly enhanced the expression of these genes in the scion (Fig. 8).

## Discussion

Many horticultural plants require grafting for asexual propagation. As grafting is typically performed using different species within the same genus, in this study we utilized two different grafting combinations: ‘Qingzhen D1/Qingzhen D1’ and ‘QAUP-1/Qingzhen D1’. During the grafting healing process, the self-grafted combination of the same species produces callus tissue more rapidly, while this phenomenon is delayed in interspecific grafts, indicating the necessity to overcome interspecific incompatibility postgrafting. However, the healing process can still be completed within 25 days. Accompanied by the rapid growth of the scion in later stages, the long-term impact of heterologous rootstocks on the scion becomes more pronounced, and their interspecific incompatibility gradually manifests [33]. Metabolomic analysis of the early-stage differences in graft healing revealed that the proportion of differential metabolites between self-grafted and interspecific grafts exceeded 10% of the total metabolite in both cases. This indicates that there is a vigorous metabolic process occurring between the rootstock and scion after grafting. KEGG enrichment analysis revealed that the differential metabolites on the first day were mainly concentrated in amino acid metabolism pathways, suggesting that the plant needs to synthesize a large amount of new substances such as proteins to promote healing after grafting [34]. In studies of cucumber graft healing, the positive regulatory role of the amino acid metabolic pathway has also been demonstrated [28].

Furthermore, Sugars, as respiratory substrates, are essential for energy production and the synthesis of macromolecules [35]. KEGG enrichment analysis revealed that the ‘starch and sucrose metabolism’, ‘fructose and mannose metabolism’, and ‘galactose metabolism’ pathways were enriched during the grafting healing process. Specifically, the contents of fructose, D-mannitol, GDP-glucose, 6P-mannitol, 1P-mannitol, stachyose, trehalose, 6P-trehalose, sucrose, and 1P-glucose decreased at the grafting interface, indicating that grafting healing is a sugar consumption process. Furthermore, during the graft union healing process in melon/pumpkin grafts, the concentrations of sugars such as sucrose, glucose, and fructose exhibit an initial increase followed by a subsequent decrease. This phenomenon suggests that plants enhance graft healing by transiently elevating carbohydrate levels to meet metabolic demands [36]. Similarly, this hypothesis has been corroborated in apple graft healing research, where sugar accumulation correlates with callus formation and vascular reconnection [37]. In pear grafting studies, sugars have been shown to promote grafting compatibility, with significant alterations in sugar metabolism profiles observed when pear scions were grafted onto different rootstocks [7, 38]. Notably, our previous comparative analysis of self-grafted and interspecific graft combinations revealed significant enrichment of genes related to carbohydrate metabolic pathways postgrafting, particularly in the early healing stages [39].

Neutral invertase can catalyze the breakdown of fructose into glucose. In the early stages, the expression level of the neutral invertase gene in the interspecific grafting combination was lower than that in the self-grafting combination, and the glucose content in the interspecific grafting combination was also lower than that in the self-grafting combination. Neutral invertase plays a pivotal role in the graft union, as the sugar metabolism fluctuations it mediates are closely associated with the establishment of graft resistance mechanisms [40, 41]. Furthermore, its downstream metabolic product glucose actively promotes the healing process of graft unions [36]. This may be one of the reasons why its healing ability was lower than that of the self-grafting combination in the early stages.

Notably, through analysis of the ‘mannose and fructose’ metabolism pathway, sorbitol exhibited opposite expression trends in the self-grafting and interspecific grafting combinations. In the interspecific grafting combination, sorbitol content increased after grafting, while it decreased in the self-grafting combination. This suggests that sorbitol may be an important metabolite contributing to the differences in healing ability between self-grafting and interspecific grafting combinations. Spatial metabolomics was used to explore the spatial distribution of sorbitol. In self-grafted combinations, sorbitol accumulates above the graft union, whereas during later stages, its content shows a marked increase in interspecific grafts. In pears, intense metabolic activity in the upper part of the scion after grafting. As grafting healing progressed, the proportions of differential metabolites in each region decreased to 8%, 7%, 8%, and 12%, respectively, suggesting that the early stages of grafting may be more critical for healing. More differential metabolites were present in the interspecific grafting combination 9 days after grafting, indicating more frequent material exchanges within the tissues during the early stages of healing in the interspecific grafting combination. This may be because, during the formation of intraspecific grafting unions, the scion and rootstock can recognize adjacent tissues and activate wound healing mechanisms different from those in separated tissues, while interspecific grafting tissues may need to first overcome interspecific barriers [13, 19]. Recent studies have shown that asymmetric expression above and below the grafting interface may be key to grafting fusion. For example, transcriptome dynamics analysis has shown that many genes related to cambium, phloem, and xylem development, and sugar response are expressed at higher levels on one side than the other at the scion of homografted Arabidopsis, and this asymmetry disappears after vascular tissue reconnection [6, 19]. This also suggests that the contributions to healing after grafting are unequal, with the scion possibly playing a more important role in pear. Therefore, the accumulation of sorbitol in early-stage self-grafted combinations suggests its crucial role in graft union healing. However, to date, functional studies investigating sorbitol’s specific role in the graft healing process remain scarce.

Therefore, through exogenous sorbitol treatment, it was found that sorbitol could promote rapid union of the graft, resulting in a higher bonding strength and tighter connection between the rootstock and the scion. In the sorbitol-treated group, the fluorescent signal diffused more rapidly into the rootstock, and the fluorescent area was also larger, indicating that sorbitol promoted the rapid differentiation of vascular tissues. Histological observations revealed that sorbitol stimulated cell proliferation, leading to the production of abundant callus tissue, accelerated cell differentiation, and earlier formation of vascular bundles compared to untreated grafts. Research in plant graft healing has demonstrated that the early formation of vascular bundles is a critical determinant for successful graft union establishment [18]. These results suggest that sorbitol positively promotes the grafting healing process.

Expression analysis showed that sorbitol upregulated the expression of genes related to cambium, procambium, and xylem differentiation, including *PbTMO6*, *PbPXY*, *PbPLL1*, *PbOPS*, *PbNEN4*, *PbCES1*, and *PbIRX3*, particularly in the upper region of the scion. During the cell differentiation process after grafting, genes regulating procambium, cambium, and xylem differentiation were sequentially activated over time. Sorbitol not only increased the relative expression levels of these genes but also rapidly activated genes related to xylem formation, procambium, cambium, and cell division, such as *PbTMO6*, *PbPLL1*, *PbOPS*, *PbIRX3*, and *PbH3.1*, with expression peaks occurring earlier compared to the control group. These genes have been demonstrated to be closely associated with plant wound healing and tissue regeneration [42, 43].

This further demonstrates that sorbitol can promote cell division and differentiation by rapidly activating these processes, facilitating the formation of cambium and vascular tissues, connecting the rootstock and scion, and accelerating the grafting healing process.

## Conclusion

**Figure 9:**
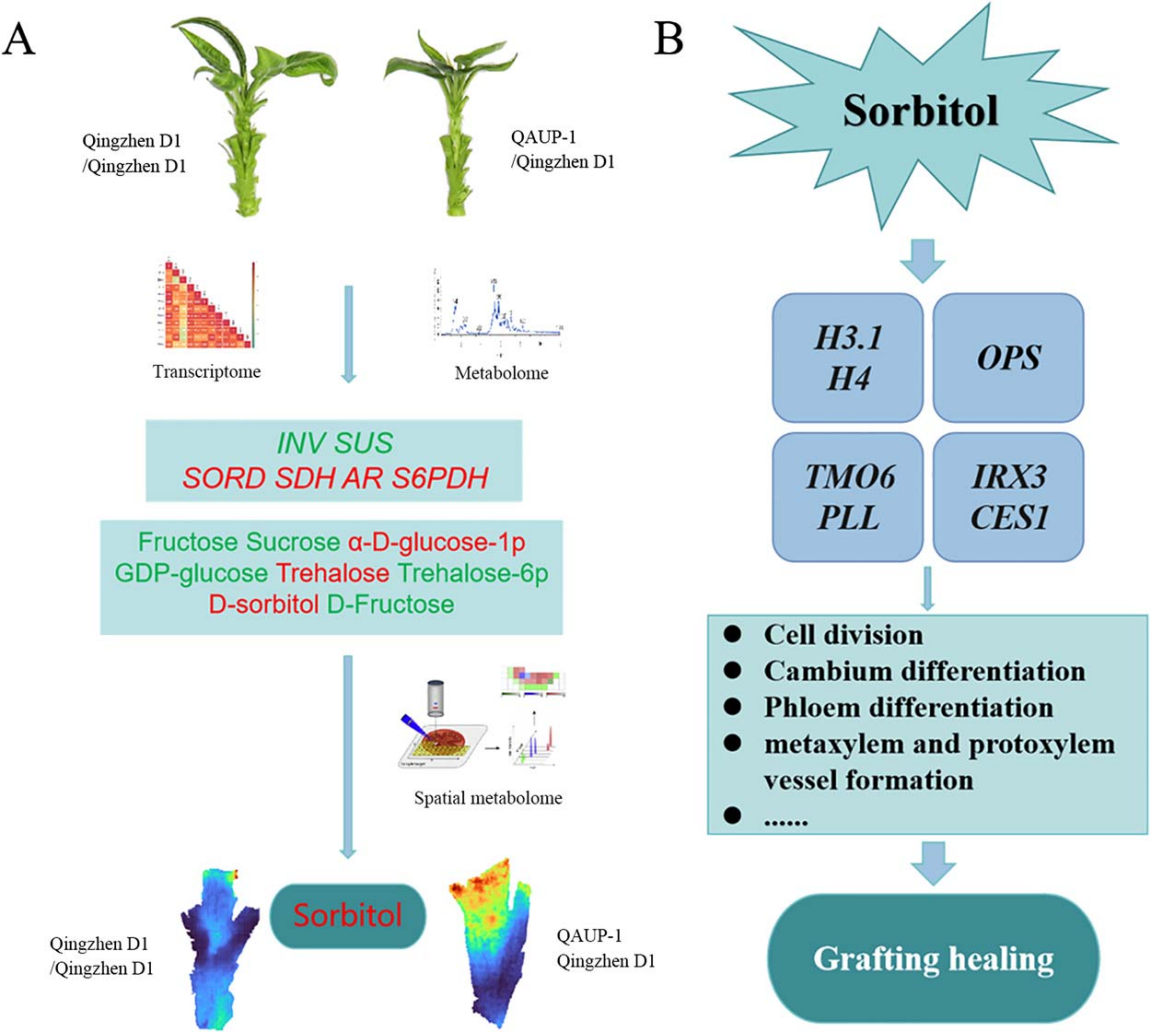
Multi-omics analysis of glucose metabolism and the mode of action of sorbitol in graft healing. (A) The combined analysis of transcriptomics and metabolomics revealed changes in glucose metabolism in the homograft combination ‘Qingzhen D1/Qingzhen D1’ and the heterograft combination ‘QAUP-1/Qingzhen D1’. *INV* and *SUS* genes were downregulated in the heterograft combination, while fructose and sucrose content were lower than in the homograft combination. The synthesis gene of sorbitol was upregulated, and the content of D-sorbitol increased. Spatial metabolomics analysis showed that heterozygosity promoted the accumulation of sorbitol in the scion. Red color words represents upregulation in heterograft, while green color words represents downregulation inheterograft. (B) Exogenous sorbitol promotes early high expression of genes such as cell division, cambium differentiation, phloem differentiation, and metaxylem and protoxylem vessel formation, promoting graft healing.

In this study, we utilized two grafting combinations, ‘Qingzhen D1/Qingzhen D1’ and ‘QAUP-1/Qingzhen D1’, and through metabolomics and transcriptomics analyses, we identified that sucrose metabolism and sorbitol metabolism play crucial roles in the grafting process. Using spatial metabolomics, we determined that sorbitol is primarily produced and functions in the scion of the graft (Fig. 9A). After autografting, sorbitol accumulates early and induces high expression and rapid response of genes related to cell division, cambium, procambium, and vascular bundles. This promotes the formation and differentiation of callus tissue at the graft interface into vascular tissues, thereby facilitating the grafting healing process (Fig. 9B). This study provides a theoretical foundation for understanding the changes and roles of sugar metabolism during grafting, as well as the application of sorbitol in the grafting process.

## Materials and methods

### Plant materials

‘Qingzhen D1’ (*Pyrus communis* Linn × *Pyrus bretschneideri* Rehd) and ‘QAUP-1’ (*Pyrus ussuriensis* Maxim) were selected as the experimental materials, both of which were provided by the pear germplasm innovation and improvement team at Qingdao Agricultural University. Two different grafting combinations were set up, both using ‘Qingzhen D1’ as the rootstock, with ‘QAUP-1’ and ‘Qingzhen D1’ as the scions, respectively, denoted as ‘Qingzhen D1/Qingzhen D1’ and ‘QAUP-1/Qingzhen D1’. Samples were taken at 1 day (rootstock separation stage), 5 days (callus formation stage), 9 days (cambium differentiation stage), 15 days (intermediate stage of differentiated vascular tissue), and 25 days (vascular bundle connection stage) after grafting.

Using the grafting combination of Qingzhen D1/Qingzhen D1 as the test material, a 6% concentration of sorbitol solution was applied externally to the grafting interface, and sterile water was used as the control. Samples were taken at 1, 3, 5, 7, 9, 15, and 25 days after grafting for morphological observation experiments on the promotion of grafting healing tissue by sorbitol. The sampling site is the stem segment at the junction of the rootstock and scion at the mating interface. The sampling location should also be used for subsequent metabolomics and transcriptome sequencing (Supplementary Fig. S5A).

### Untargeted metabolomics analysis

Samples were collected from the graft unions of the two grafting combinations, ‘Qingzhen D1/Qingzhen D1’ and ‘QAUP-1/Qingzhen D1’, at 1 and 9 days after grafting (Supplementary Fig. S6). Each treatment was repeated six times, and the sampling site was the graft union where the rootstock and scion were connected. Tissue samples (100 mg) were reconstituted in prechilled 80% methanol solution. Aliquots of the resulting supernatant underwent dilution to achieve a 53% methanol concentration using LC–MS-grade water. Following centrifugation at 15 000 rpm for 20 minutes at 4°C, the processed samples were subjected to ultra-high-performance liquid chromatography–tandem mass spectrometry (UHPLC–MS/MS) analysis. The analytical platform comprised a Thermo Fisher Vanquish UHPLC system coupled to an Orbitrap Q Exactive HF-X mass spectrometer (Thermo Fisher Scientific) operated at Novogene’s facility in Beijing, China. Chromatographic separation employed a Hypesil Gold analytical column (100 × 2.1 mm, 1.9 μm particle size). Mobile phase compositions varied between ionization modes: positive polarity analysis utilized 0.1% formic acid in water (eluent A) and methanol (eluent B), while negative polarity analysis employed 5 mM ammonium acetate buffer (pH 9.0, eluent A) and methanol (eluent B). Mass spectrometric detection parameters included: dual polarity operation (positive/negative switchable mode), 3.5 kV spray voltage, 320°C capillary temperature, 35 psi sheath gas flow, 10 l/min auxiliary gas flow, 60% S-lens radiofrequency level, and 350°C auxiliary gas heater temperature. Metabolic features were annotated through comparative analysis against the KEGG pathway database (https://www.genome.jp/kegg/pathway.html).

### Spatial metabolomics analysis

Samples were collected from the *in vitro* grafted seedlings of ‘Qingzhen D1/Qingzhen D1’ and ‘QAUP-1/Qingzhen D1’ at 1 day (grafting start period) and 25 days (grafting formation period) after grafting for spatial metabolomics measurement. Each sample was repeated three times, and the sampling site was the graft union where the rootstock and scion were connected. The samples were embedded in a freezing embedding medium (Supplementary Fig. S5B).

Tissue specimens were sectioned at 30 μm thickness using a Leica CM1950 cryostat system (Leica Microsystems GmbH, Wetzlar, Germany) maintained at −20°C. The resulting sections were transferred in batches onto indium tin oxide (ITO)-coated electrically conductive microscopy slides. Following complete desiccation, matrix deposition was performed using an HTX TM spray coating apparatus (Bruker Daltonics, Germany) to apply 2,5-dihydroxybenzoic acid (DHB) solution (15 mg/ml dissolved in 90:10 acetonitrile:water). Spray parameters included a 60°C nozzle temperature, 0.12 ml/min infusion rate, and 5 psi nitrogen gas pressure. Mass spectrometric imaging was conducted in positive ionization mode with data acquisition spanning m/z 50–1300 Da. Spatial resolution for tissue analysis was configured at 50 μm per pixel, with each pixel acquiring 400 laser irradiations to generate composite mass spectra. MALDI mass spectra were normalized with the Root Mean Square, and the signal intensity in each image was shown as the normalized intensity. MS/MS fragmentations performed on the timsTOF flex MS system in the MS/MS mode were used for further detailed structural confirmation of the identified metabolites.

### Transcriptomic analysis

Samples were collected from two grafting combinations, ‘Qingzhen D1/Qingzhen D1’ and ‘QAUP-1/Qingzhen D1’, at 1, 9, 15, and 25 days postgrafting (Supplementary Fig. S6). Each treatment was repeated three times, with the sampling site located at the graft union where the rootstock and scion are connected. The samples were stored at −80°C for sequencing, following the methods described by Ji *et al* [39]. The Mapping values of sequencing sequences are all >70%, indicating that the sequencing data has high quality, the mapping is qualified, and the transcriptome data information can be used for subsequent analysis (Supplementary Fig. S7; Supplementary Table S2).

### Histomorphological observation

The samples were fixed in Formalin-Aceto-Alcohol fixative for 24 h, then dehydrated in 50%, 75%, 95%, and 100% ethanol for 60 min each. Subsequently, they were completely decolorized in xylene solution before being embedded in paraffin. The materials were sectioned into 10-μm-thick transverse sections using a paraffin microtome. After dewaxing and rehydration in xylene and ethanol, the sections were stained with Safranin O and Fast Green for 10 min, mounted with resin, and observed under a microscope (Leica DM2500).

### Fluorescence-staining observation

A sharp single-edged razor blade was used to nick the leaves of the scion, and the leaves were placed in a centrifuge tube containing 5(6)-Carboxyfluorescein diacetate (CFDA) solution, allowing the CFDA to be absorbed from the leaf vascular bundles into the rootstock. After 24 h of dark incubation, the fluorescence in the rootstock section (0.5 cm below the graft union) and the graft interface was observed under a fluorescence microscope. The initially obtained fluorescence images were imported into Image J to calculate the fluorescence area and intensity.

### Measurement of enzyme activities related to sorbitol synthesis and metabolism

Samples were taken at 1, 5, 10, 15, and 25 days after grafting. Approximately 0.1 g of tissue sample was weighed and added to 1 ml of extraction buffer. The mixture was homogenized on ice, centrifuged at 12 000 rpm for 10 min at 4°C, and the supernatant was collected and placed on ice for subsequent measurements.

Measurement of 6-phosphosorbitol dehydrogenase activity: 6-phosphosorbitol dehydrogenase catalyzes the reduction of D-glucose 6-phosphate and oxidizes reduced coenzyme II. By detecting the rate of decrease in NADPH at 340 nm, the enzyme activity of 6-phosphosorbitol dehydrogenase can be determined. The activity was measured using a 6-Phosphosorbitol Dehydrogenase Activity Kit (Shanghai Tongwei Biotechnology Co., Ltd., Catalog No.: ADS-F-TDX057).

Measurement of AR activity: plant AR was added, followed by incubation with HRP-labeled AR antibodies to form an antibody antigen enzyme-labeled antibody complex. After washing, the substrate TMB (3,3′,5,5′-tetramethylbenzidine) was added for color development, and the absorbance was measured at 450 nm. The AR activity concentration in the sample was calculated using a standard curve. The activity was measured using an Aldose Reductase Activity Kit (Shanghai Tongwei Biotechnology Co., Ltd., Catalog No.: MM-36124O1).

Measurement of NADP^+^-sorbitol dehydrogenase (NADP^+^-SDH) activity: NADP^+^-SDH catalyzes the dehydrogenation of sorbitol to glucose, simultaneously reducing NADP^+^ to NADPH. The enzyme activity of NADP^+^-SDH can be calculated by measuring the rate of increase in absorbance at 340 nm. The activity was measured using an NADP^+^-Sorbitol Dehydrogenase Kit (Shanghai Tongwei Biotechnology Co., Ltd., Catalog No.: ADS-W-TDX054).

Measurement of NAD^+^-sorbitol dehydrogenase (NAD^+^-SDH) activity: NAD^+^-SDH catalyzes the dehydrogenation of sorbitol to fructose, simultaneously reducing NAD^+^ to NADH. The enzyme activity of NAD^+^-SDH can be calculated by measuring the rate of increase in absorbance at 340 nm. The activity was measured using a NAD^+^-Sorbitol Dehydrogenase Kit (Shanghai Tongwei Biotechnology Co., Ltd., Catalog No.: ADS-W-TDX055).

### Sugar content determination

Take 0.3 g of processed sample into a centrifuge tube, add 1.5 ml of 50% acetonitrile, incubate at 70°C for 20 min, centrifuge at 9000 rpm for 15 min, take the supernatant, add 1.5 ml of 50% acetonitrile, incubate at 70°C for 20 min, centrifuge at 9000 rpm for 15 min, and dilute to 4 ml. Used for ion chromatography determination. Chromatographic column: EICOEE BD Amide 3.5 μm 150*4.6 mm; Flow rate: 1.0 ml/min; Column temperature: 50°C; Detector: evaporative light detector; Detector gain: 100; Filter time constant: 0.2; Gas pressure: 30 psi; Drift tube: 50°C; Injection volume: 10 μl; Mobile phase: A 0.2% triethylamine aqueous solution B acetonitrile. Elution procedure (Supplementary Table S3).

### Real-time quantitative polymerase chain reaction analysis

Samples were taken at 1, 3, 5, 7, 9, 15, 20 and 30 days after grafting. Using the Roche 480 real-time PCR system (Basil, Switzerland) in standard mode, real-time quantitative polymerase chain reaction (qRT-PCR) analysis was conducted with ChamQ SYBR Color qPCR Master Mix. The qRT-PCR amplification program was as follows: 95°C for 5 min, followed by 45 cycles of 95°C for 15 s, 60°C for 30 s, and 72°C for 30 s. Using the pear actin gene as an internal control. Relative gene expression levels were determined via the 2^−ΔΔCT^ method [44], and each sample was analyzed in triplicate copies. The primers are listed in Supplementary Table S4.

### Statistical analysis

Graphs were constructed using GraphPad Prism 9.5. Analysis of variance (ANOVA) was performed using the SPSS 23.0 statistical software package (IBM SPSS Statistics, Chicago, IL, USA), and means were compared using Duncan’s test (*P* < 0.05).

## Supplementary Material

Web_Material_uhaf168

## Data Availability

The raw sequence data of RNA-seq was deposited at National Center for Biotechnology Infommation (NCBI, https://www.ncbi.nlm.nih.gov/bioproject/) under accession number GSE190654. All data generated or analyzed during this study are included in the manuscript and supporting files (Figs S1–S7; Tables S1–S4).
