# Supplementary material for: Sorbitol promotes the graft healing process in pears
Source: Hortic Res. 2025 Jun 25;12(9):uhaf168. doi: 10.1093/hr/uhaf168 (PMC12373972; doi:10.1093/hr/uhaf168)
Supplement: Web_Material_uhaf168 [file web_material_uhaf168.zip › FigureS1-S7.docx]

Figure S1


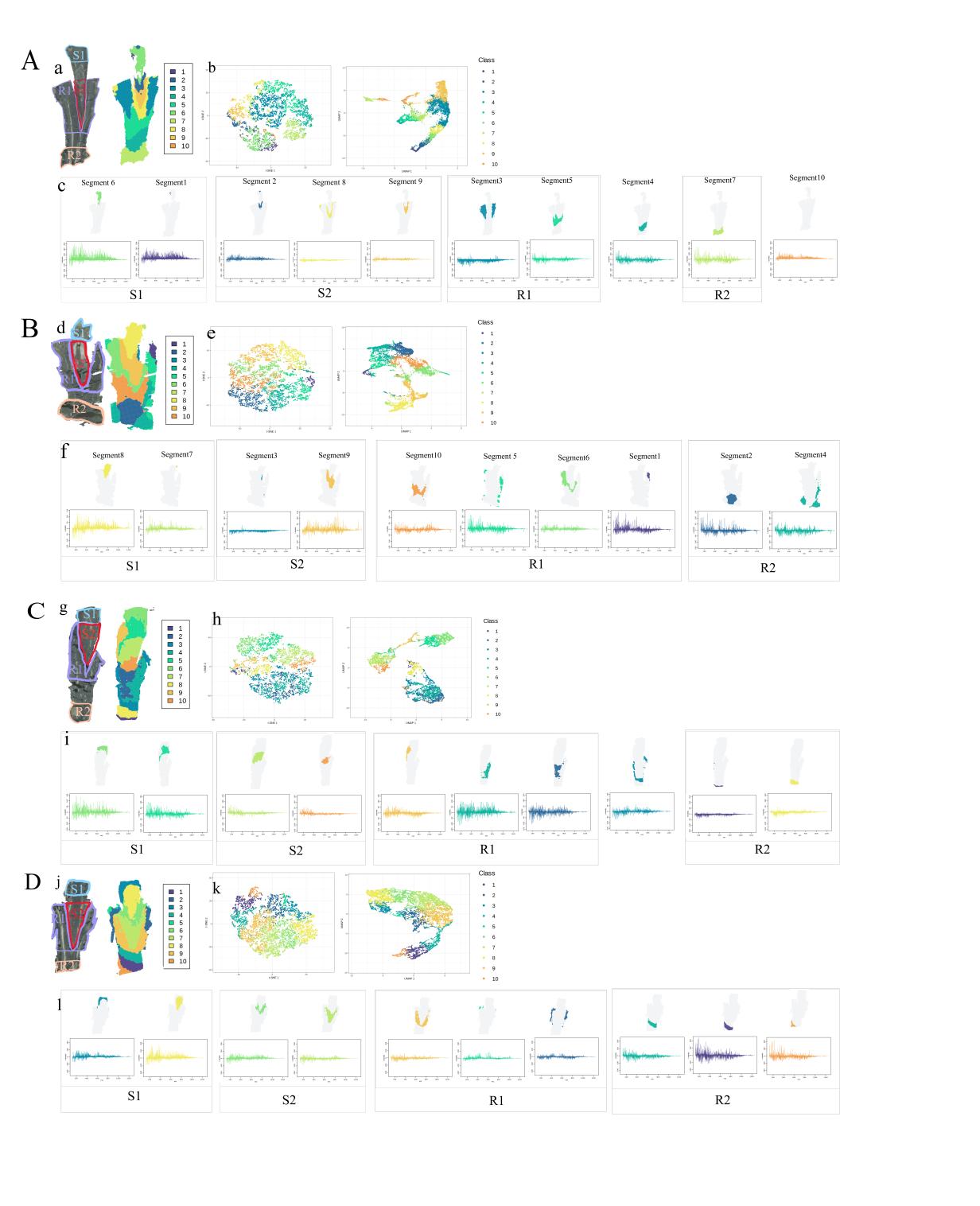


Figure S1 (A) The color coverage area of the scion and rootstock after 1 day of grafting with the combination of QAUP-1/Qingzhen D1. a,d,g,j:Spatial segmentation map : The different colors in the figure represent different regions of spatial segmentation; b,e,h,k:T-SNE 2D plan view:Different points represent various pixels on the plane of mass spectrometry imaging scanned tissue samples, and the closer the points are, the more similar the metabolite abundance characteristics are. The color of the points corresponds to the spatial segmentation results; c,f,i,l:t-statistics and spatial distribution of regional characteristic metabolites:The above figure shows the corresponding area for spatial segmentation analysis, and the following figure shows the t-statistic of each target peak in the corresponding area, with m/z on the x-axis and t-statistic on the y-axis.(B) The color coverage area of the scion and rootstock after 25 day of grafting with the combination of QAUP-1/Qingzhen D1. (C) The color coverage area of the scion and rootstock after 1 day of grafting with the combination of Qingzhen D1/Qingzhen D1. (D) The color coverage area of the scion and rootstock after 25 day of grafting with the combination of Qingzhen D1/Qingzhen D1.

Figure S2


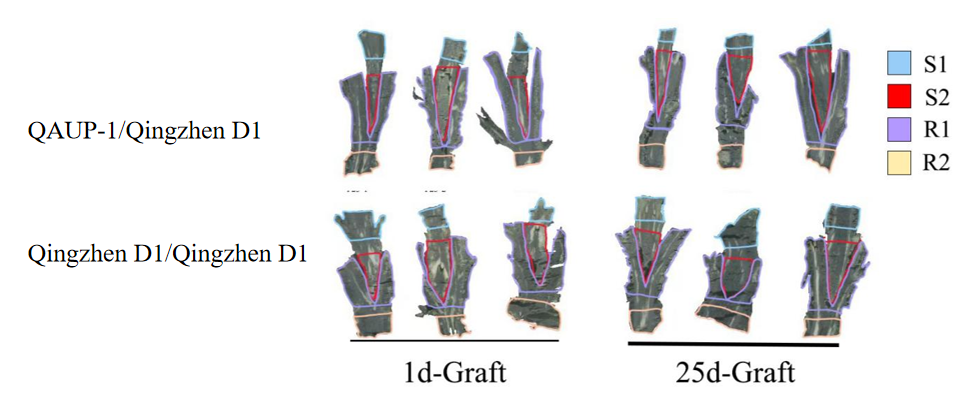


Figure S2. Scan of tissue section. S1: represents the upper part of the scion; S2: represents the lower part of the scion; R1: represents the upper part of the rootstock; R2: represents the lower part of the rootstock.

Figure S3


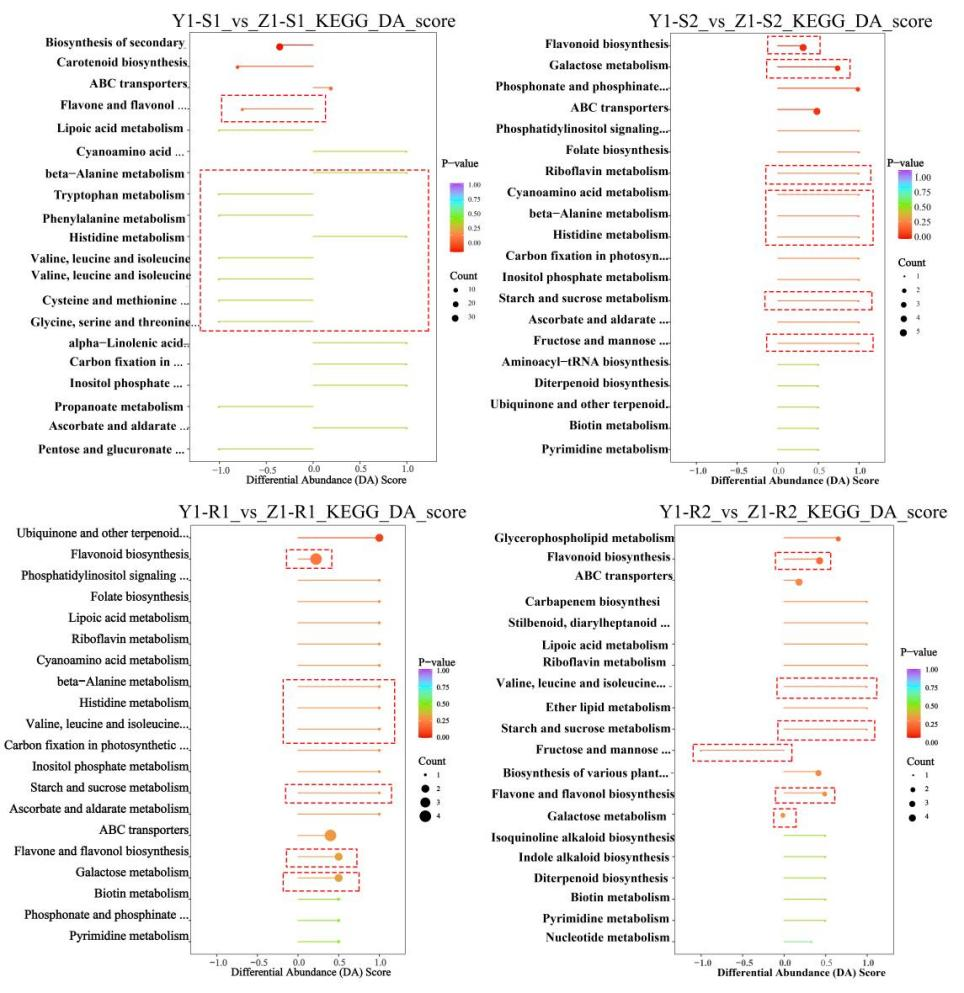


Figure S3. KEGG enrichment analysis of differential gene pathways in Y1 vs Z1.

Figure S4


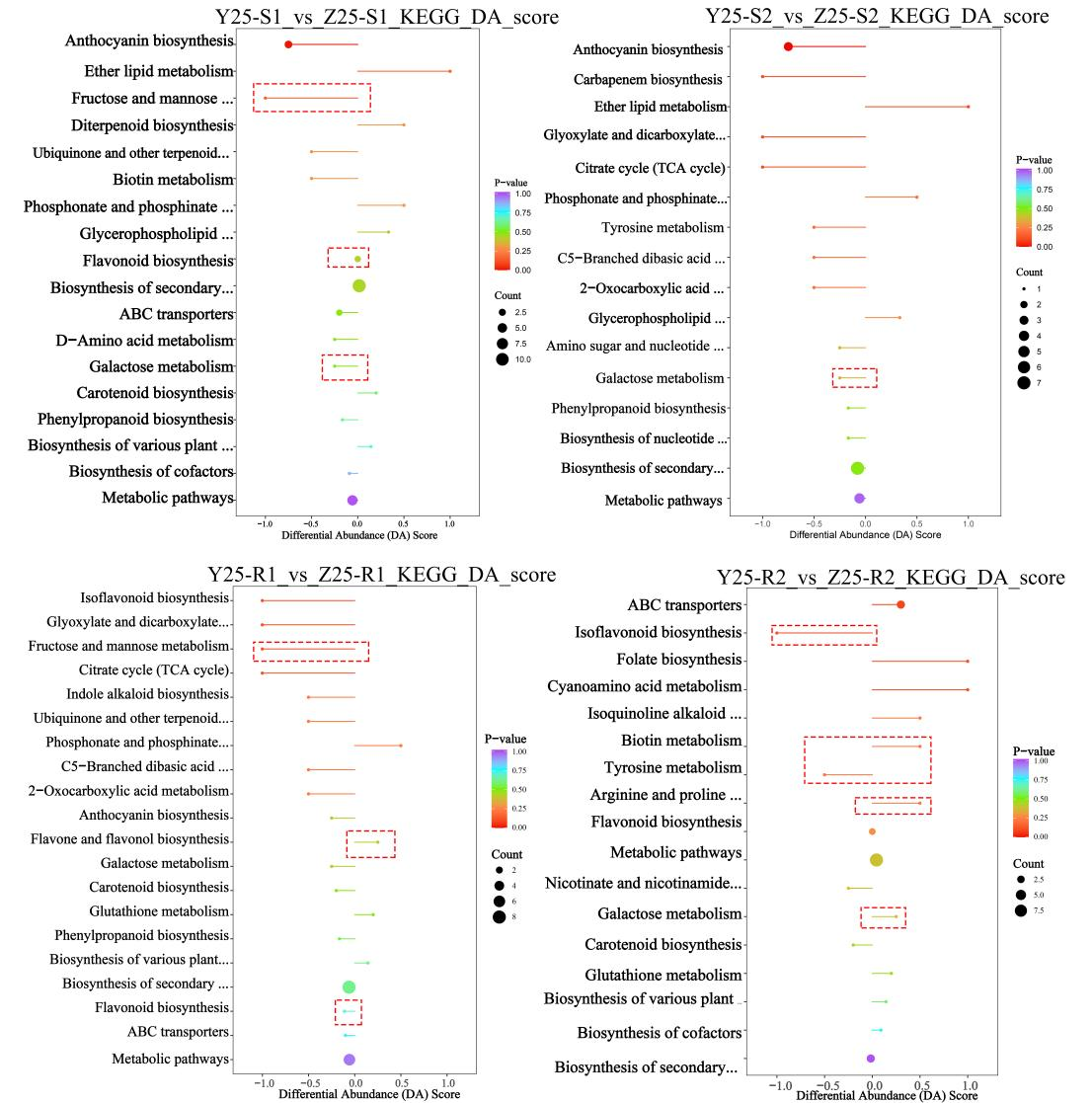


Figure S4. KEGG enrichment analysis of differential gene pathways in Y25 vs Z25.

Figure S5


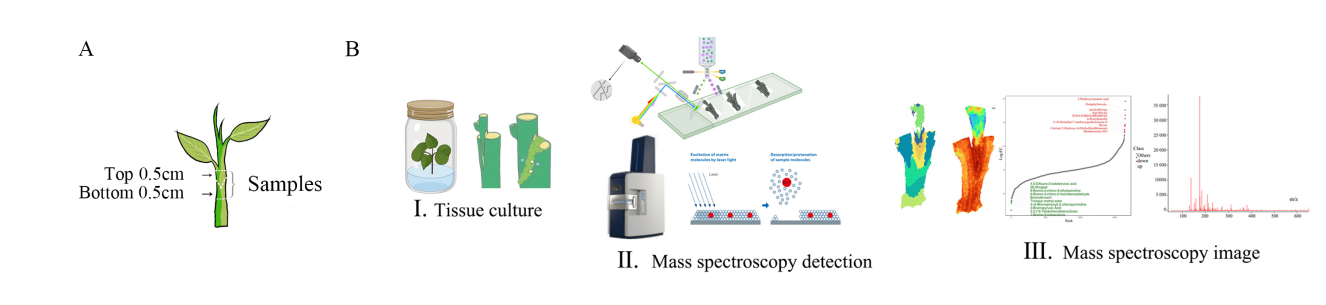


**Figure** S5**. Sampling sites and spatial metabolic profiling. (A) The location of the sampling sample. (B) Procedure for spatial metabolomics experiment. Ⅰ:Tissue culture; Ⅱ:Mass spectroscopy detection, Ⅲ: Mass spectroscopy image.**

Figure S6


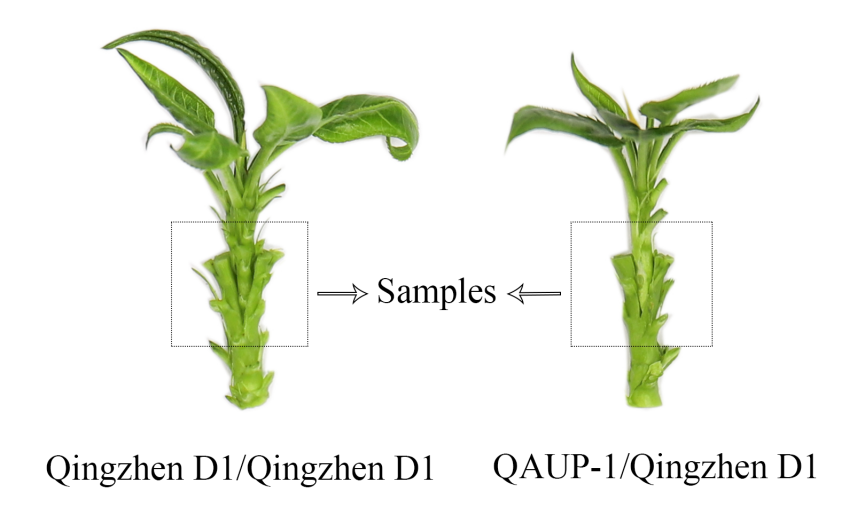


Figure S6 **Transcriptome and Metabolome Sampling Sites**

Figure S7


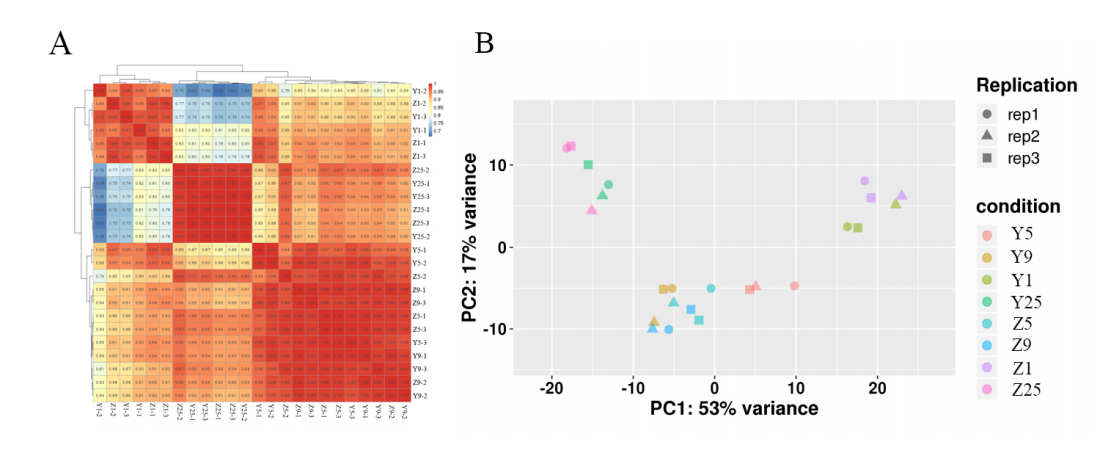


**Figure S7. Quality inspection of transcriptome samples. (A) Correlation analysis between total samples (B) PCA analysis of total sample mass spectrometry data.**
